# Supplementary material for: Bioinformatical Design and Performance Evaluation of a Nucleocapsid- and an RBD-Based Particle Enhanced Turbidimetric Immunoassay (PETIA) to Quantify the Wild Type and Variants of Concern-Derived Immunoreactivity of SARS-CoV-2
Source: Biomedicines. 2023 Jan 8;11(1):160. doi: 10.3390/biomedicines11010160 (PMC9855841; doi:10.3390/biomedicines11010160)
Supplement: Supplementary file 1 [file biomedicines-11-00160-s001.zip › biomedicines-2106354-supplementary.pdf]

Supplementary data, Wey *et al.*

Supplementary Table S1. UniProt entry numbers of primary sequences of the Nucleocapsid protein, Spike protein, and the Spike-RBD protein of SARS-CoV-2 and the other human infecting coronaviruses (SARS-CoV, MERS-CoV, HCoV-OC43, HCoV-HKU1, HCoV-NL63, and HCoV-229E). *HCoV-NL63 and HCoV-229E primary sequences involved in interaction with host ANPEP were chosen to compare with SARS-CoV-2's S-RBD.

Virus	SARS-CoV-2		
	**N protein** ***(P0DTC9)***	**S protein** ***(P0DTC2)***	**S-RBD** ***(P0DTC2, aa 319–541)***
SARS-CoV	P59595	P59594	P59594, aa 306–527
MERS-CoV	R9UM87	W6A028	W6A028, aa 394–586
HCoV-OC43	P33469	P36334	P36334, aa 318–624
HCoV-HKU1	Q5MQC6	Q5MQD0	Q5MQD0, aa 310–624
HCoV-NL63	Q6Q1R8	Q6Q1S2	Q6Q1S2, aa 598–728 *Interaction with host ANPEP
HCoV-229E	P15130	P15423	P15423, aa 417–547 *Interaction with host ANPEP

Supplementary Table S2. S-RBD antigen concentration of the respective variants (wild type, *Alpha, Beta, Gamma, Kappa, Delta, Omicron*) in the reaction [ng] and related quantified anti-SARS-CoV-2 concentration [BAU/mL] after quenching.

Antigen concentration [ng]	wild type	*Alpha*	*Beta*	*Gamma*	*Kappa*	*Delta*	*Omicron*
	anti-SARS-CoV-2 concentration [BAU/mL]						
100.00	0.61	0.74	0.45	0.42	0.58	0.34	3.11
80.00	0.83	0.60	0.44	0.55	0.55	0.26	3.40
60.00	1.47	0.79	0.66	0.80	0.91	1.22	3.51
40.00	1.36	0.92	1.27	1.03	1.30	1.14	4.60
20.00	2.86	2.27	1.87	1.52	2.50	2.18	5.65
10.00	4.52	3.74	3.10	3.12	3.67	3.70	9.14
5.00	9.33	7.69	7.20	7.42	7.28	7.55	17.71
2.50	15.18	12.89	11.67	12.16	12.52	12.44	21.95
0.00	26.86	26.86	26.86	26.86	26.86	26.86	26.86

Supplementary Table S3. S-RBD antigen concentration of the respective variants (wild type, *Alpha, Beta, Gamma, Kappa, Delta, Omicron*) in the reaction [ng] and resulting recovery [%] related to a non-quenched blank sample (anti-SARS-CoV-2 concentration after quenching with 0 ng antigen refers to 100% recovery).

Antigen concentration [ng]	wild type	*Alpha*	*Beta*	*Gamma*	*Kappa*	*Delta*	*Omicron*
	recovery [%]						
100.00	2.3	2.8	1.7	1.6	2.2	1.3	11.6
80.00	3.1	2.2	1.7	2.1	2.0	1.0	12.7
60.00	5.5	2.9	2.4	3.0	3.4	4.6	13.1
40.00	5.1	3.4	4.7	3.8	4.9	4.3	17.1
20.00	10.7	8.5	6.9	5.7	9.3	8.1	21.0
10.00	16.8	13.9	11.5	11.6	13.7	13.8	34.0
5.00	34.7	28.6	26.8	27.6	27.1	28.1	65.9
2.50	56.5	48.0	43.4	45.3	46.6	46.3	81.7
0	100.0	100.0	100.0	100.0	100.0	100.0	100.0

Supplementary Table S4. S-RBD antigen concentration of the *Omicron* variant in the reaction [ng] and resulting SARS-CoV-2 concentration [BAU/mL] and recovery [%] related to a non-quenched blank sample (anti-SARS-CoV-2 concentration after quenching with 0 ng antigen (26.86 BAU/mL) refers to 100% recovery).

Antigen concentration [ng]	*Omicron*	
	anti-SARS-CoV-2 concentration [BAU/mL]	recovery [%]
1000.00	1.55	5.8
500.00	1.04	3.9

Supplementary Table S5. (**a**) Diagnostic table (2x2) and (**b**) calculated sensitivity and specificity of the S-RBD-based PETIA. Results generated with MedCalc Statistical Software version 18.10.2 (MedCalc Software bvba, Ostend, Belgium; http://www.medcalc.org; 2018, accessed on 29 November 2021).

(**a**)

	S-RBD		
	Disease Present	Disease Absent	
Test Positive	40	1	41
Test Negative	3	51	54
	43	52	

(**b**)

Sensitivity	93.023%	80.939% to 98.537%
Specificity	98.077%	89.745% to 99.951%
AUC	0.956	0.892 to 0.987
Positive Likelihood Ratio	48.372	6.932 to 337.549
Negative Likelihood Ratio	0.071	0.024 to 0.212
Disease prevalence	45.263%	35.021% to 55.809%
Positive Predictive Value	97.561%	85.146% to 99.643%
Negative Predictive Value	94.444%	85.083% to 98.065%
Accuracy	95.789%	89.569% to 98.841%

Supplementary Table S6. (**a**) Diagnostic table (2x2) and (**b**) calculated sensitivity and specificity of the N protein-based PETIA. Results generated with MedCalc Statistical Software version 18.10.2 (MedCalc Software bvba, Ostend, Belgium; http://www.medcalc.org; 2018, accessed on 29 November 2021).

(**a**)

	N protein		
	Disease Present	Disease Absent	
Test Positive	43	41	84
Test Negative	0	11	11
	43	52	

(**b**)

Sensitivity	100.000%	91.779% to 100.000%
Specificity	21.154%	11.061% to 34.704%
AUC	0.606	0.500 to 0.705
Positive Likelihood Ratio	1.268	1.102 to 1.460
Negative Likelihood Ratio	0.000	
Disease prevalence	45.263%	35.021% to 55.809%
Positive Predictive Value	51.190%	47.673% to 54.696%
Negative Predictive Value	100.000%	
Accuracy	56.842%	46.277% to 66.969%

Supplementary Table S7. Values of the negative and positive COVID-19 samples, analyzed by the N-based Roche Elecsys® Anti-SARS-CoV-2 CLIA, the S-RBD- and N-based PETIA. Samples indicated with < LOQ (Limit of Quantitation), were considered as 0.

Positive samples			Negative samples		
Elecsys® Anti-SARS-CoV-2 test [CoI]	S-RBD based PETIA [BAU/mL]	N protein-based PETIA [BAU/mL]	Elecsys® Anti-SARS-CoV-2 test [CoI]	S-RBD based PETIA [BAU/mL]	N protein based PETIA [BAU/mL]
43.30	99.75	109.74	< LOQ	< LOQ	< LOQ
58.40	84.51	136.23	< LOQ	4.86	7.19
67.10	69.72	133.86	< LOQ	< LOQ	< LOQ
42.20	95.19	135.69	< LOQ	5.58	20.07
13.90	75.72	50.07	< LOQ	< LOQ	5.73
110.00	87.87	168.75	< LOQ	< LOQ	< LOQ
6.22	10.05	32.55	< LOQ	4.26	6.82
8.55	6.03	81.78	< LOQ	< LOQ	< LOQ
89.10	92.10	164.46	< LOQ	< LOQ	< LOQ
101.00	30.87	168.27	< LOQ	< LOQ	11.15
8.60	6.78	39.27	< LOQ	< LOQ	< LOQ
81.90	101.25	148.77	< LOQ	< LOQ	24.21
18.60	59.49	90.51	< LOQ	< LOQ	7.85
15.00	40.41	61.14	< LOQ	< LOQ	< LOQ
97.90	106.02	172.71	< LOQ	< LOQ	< LOQ
30.90	93.57	97.05	< LOQ	< LOQ	42.07
97.80	89.61	154.23	< LOQ	< LOQ	43.57
103.00	94.20	174.00	< LOQ	< LOQ	< LOQ
111.00	76.29	136.41	< LOQ	< LOQ	44.55
109.00	90.99	163.44	< LOQ	6.87	42.60
32.90	86.58	140.31	< LOQ	3.45	40.78
2.41	58.89	21.93	< LOQ	< LOQ	43.99
84.70	23.70	158.67	< LOQ	6.39	43.21
98.10	16.02	168.06	< LOQ	< LOQ	44.51
50.80	75.24	141.33	< LOQ	< LOQ	43.96
44.90	79.41	134.55	< LOQ	< LOQ	44.26
84.10	93.60	166.95	< LOQ	< LOQ	46.45
57.90	97.77	153.66	< LOQ	< LOQ	43.76
49.10	16.95	105.15	< LOQ	< LOQ	44.15
60.30	88.95	167.07	< LOQ	4.56	47.04
24.80	64.05	81.78	< LOQ	< LOQ	44.38
81.00	87.54	161.10	< LOQ	< LOQ	42.78
83.80	95.01	136.86	< LOQ	< LOQ	44.54
89.70	93.81	136.44	< LOQ	< LOQ	48.29
36.36	97.65	151.05	< LOQ	< LOQ	41.92
13.52	78.60	51.54	< LOQ	13.14	41.66
46.01	27.36	131.04	< LOQ	< LOQ	41.84
12.85	19.59	56.82	< LOQ	< LOQ	43.25
132.70	85.98	175.26	< LOQ	5.28	41.08
123.80	97.35	165.66	< LOQ	< LOQ	46.29
123.20	69.60	164.73	< LOQ	< LOQ	42.80
134.70	85.23	169.68	< LOQ	3.18	42.56
7.33	4.32	46.02	< LOQ	< LOQ	40.73
			< LOQ	< LOQ	40.29
			< LOQ	< LOQ	42.79
			< LOQ	< LOQ	47.08
			< LOQ	< LOQ	45.22
			< LOQ	5.28	10.85
			< LOQ	< LOQ	< LOQ
			< LOQ	< LOQ	< LOQ
			< LOQ	< LOQ	< LOQ
			< LOQ	< LOQ	4.89
